# Supplementary material for: Phytochemicals with Chemopreventive Activity Obtained from the Thai Medicinal Plant Mammea siamensis (Miq.) T. Anders.: Isolation and Structure Determination of New Prenylcoumarins with Inhibitory Activity against Aromatase
Source: Int J Mol Sci. 2022 Sep 23;23(19):11233. doi: 10.3390/ijms231911233 (PMC9570088; doi:10.3390/ijms231911233)
Supplement: Supplementary file 1 [file ijms-23-11233-s001.zip › ijms-1924807-supplementary.pdf]

Supplementary Materials:

Phytochemicals with Chemopreventive Activity Obtained from the Thai Medicinal Plant *Mammea siamensis* (Miq.) T. Anders.: Isolation and Structure Determination of New Prenylcoumarins Inhibitory Activity against Aromatase

Fenglin Luo [1], Yoshiaki Manse [1], Saowanee Chaipech [1,2], Yutana Pongpiriyadacha [3], Osamu Muraoka [1], and Toshio Morikawa [1,*]

[1] Pharmaceutical Research and Technology Institute, Kindai University, 3-4-1 Kowakae, Higashi-osaka 577-8502, Osaka, Japan
[2] Faculty of Agro-Industry, Rajamangala University of Technology Srivijaya, Thungyai, Nakhon Si Thammarat, 80240, Thailand
[3] Faculty of Science and Technology, Rajamangala University of Technology Srivijaya, Thungyai, Nakhon Si Thammarat, 80240, Thailand
* Correspondence: morikawa@kindai.ac.jp; Tel. +81-6-4307-4306; Fax: +81-6-6729-3577

Table of Contents

Figure S1. ^{1}H-NMR (800 MHz, $CDCl_3$) spectrum of mammeasin K (**1**) S2
Figure S2. ^{13}C-NMR (200 MHz, $CDCl_3$) spectrum of mammeasin K (**1**) S3
Figure S3. ^{1}H–^{1}H COSY spectrum of mammeasin K (**1**) S4
Figure S4. HSQC spectrum of mammeasin K (**1**) S5
Figure S5. HMBC spectrum of mammeasin K (**1**) S6
Figure S6. ^{1}H-NMR (800 MHz, $CDCl_3$) spectrum of mammeasin L (**2**) S7
Figure S7. ^{13}C-NMR (200 MHz, $CDCl_3$) spectrum of mammeasin L (**2**) S8
Figure S8. ^{1}H–^{1}H COSY spectrum of mammeasin L (**2**) S9
Figure S9. HSQC spectrum of mammeasin L (**2**) S10
Figure S10. HMBC spectrum of mammeasin L (**2**) S11
Figure S11. ^{1}H-NMR (500 MHz, $CDCl_3$) spectrum of mammeasin M (**3**) S12
Figure S12. ^{13}C-NMR (125 MHz, $CDCl_3$) spectrum of mammeasin M (**3**) S13
Figure S13. ^{1}H–^{1}H COSY spectrum of mammeasin M (**3**) S14
Figure S14. HSQC spectrum of mammeasin M (**3**) S15
Figure S15. HMBC spectrum of mammeasin M (**3**) S16
Figure S16. Difference NOE spectrum of mammeasin M (**3**) S17
Figure S17. ^{1}H-NMR (800 MHz, $CDCl_3$) spectrum of mammeasin N (**4**) S18
Figure S18. ^{13}C-NMR (200 MHz, $CDCl_3$) spectrum of mammeasin N (**4**) S19
Figure S19. ^{1}H–^{1}H COSY spectrum of mammeasin N (**4**) S20
Figure S20. HSQC spectrum of mammeasin N (**4**) S21
Figure S21. HMBC spectrum of mammeasin N (**4**) S22
Figure S22. ^{1}H-NMR (500 MHz, $CDCl_3$) spectrum of mammeasin O (**5**) S23
Figure S23. ^{13}C-NMR (125 MHz, $CDCl_3$) spectrum of mammeasin O (**5**) S24
Figure S24. ^{1}H–^{1}H COSY spectrum of mammeasin O (**5**) S25
Figure S25. HSQC spectrum of mammeasin O (**5**) S26
Figure S26. HMBC spectrum of mammeasin O (**5**) S27
Figure S27. Lineweaver-Burk plots of the inhibition of human recombinant aromatase activity by **1**, **4–11**, **16**, **17**, **23–25**, **28**, **33**, **38**, and **39** S28

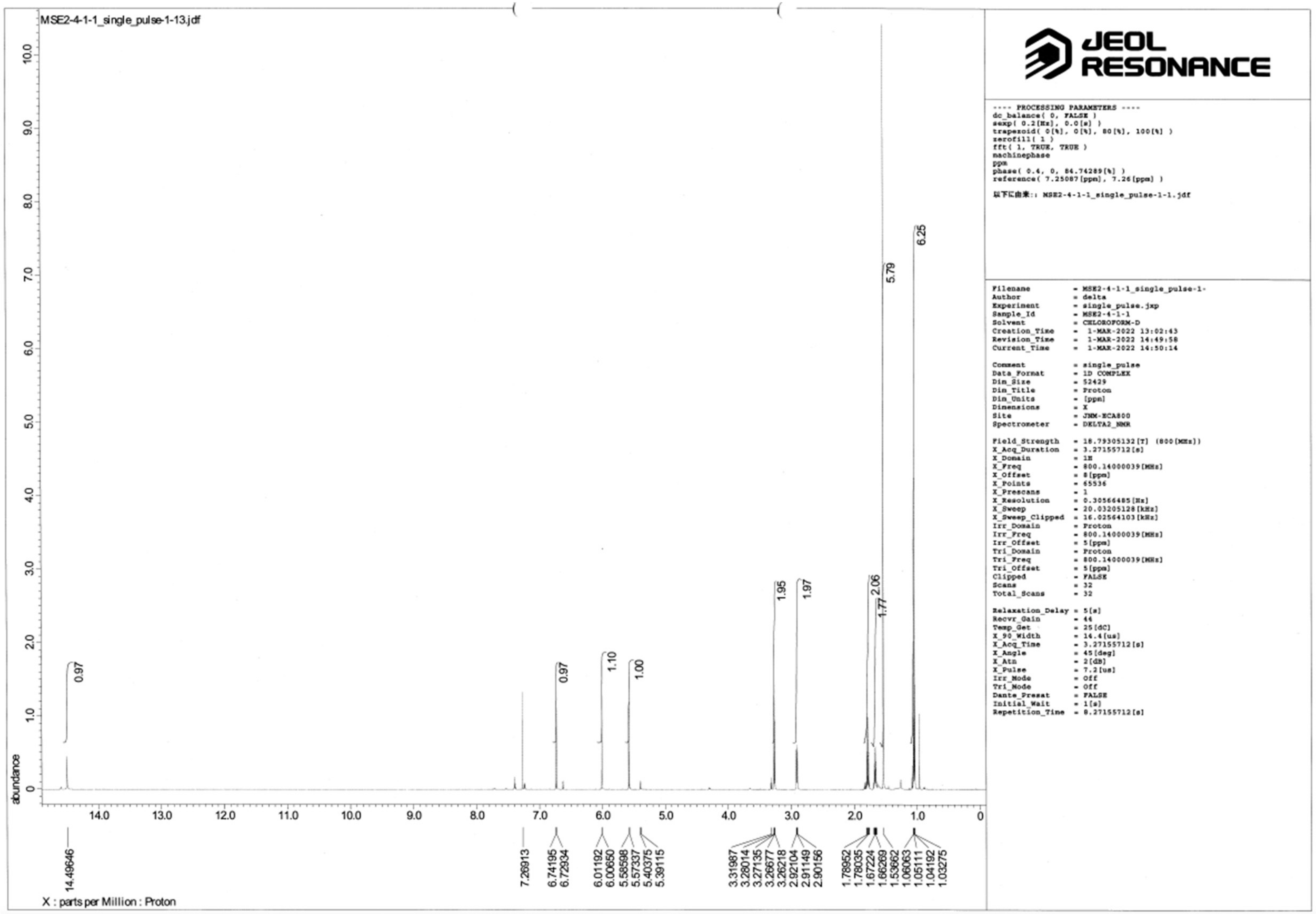

Figure S1. ^{1}H-NMR (800 MHz, $CDCl_3$) spectrum of mammeasin K (**1**)

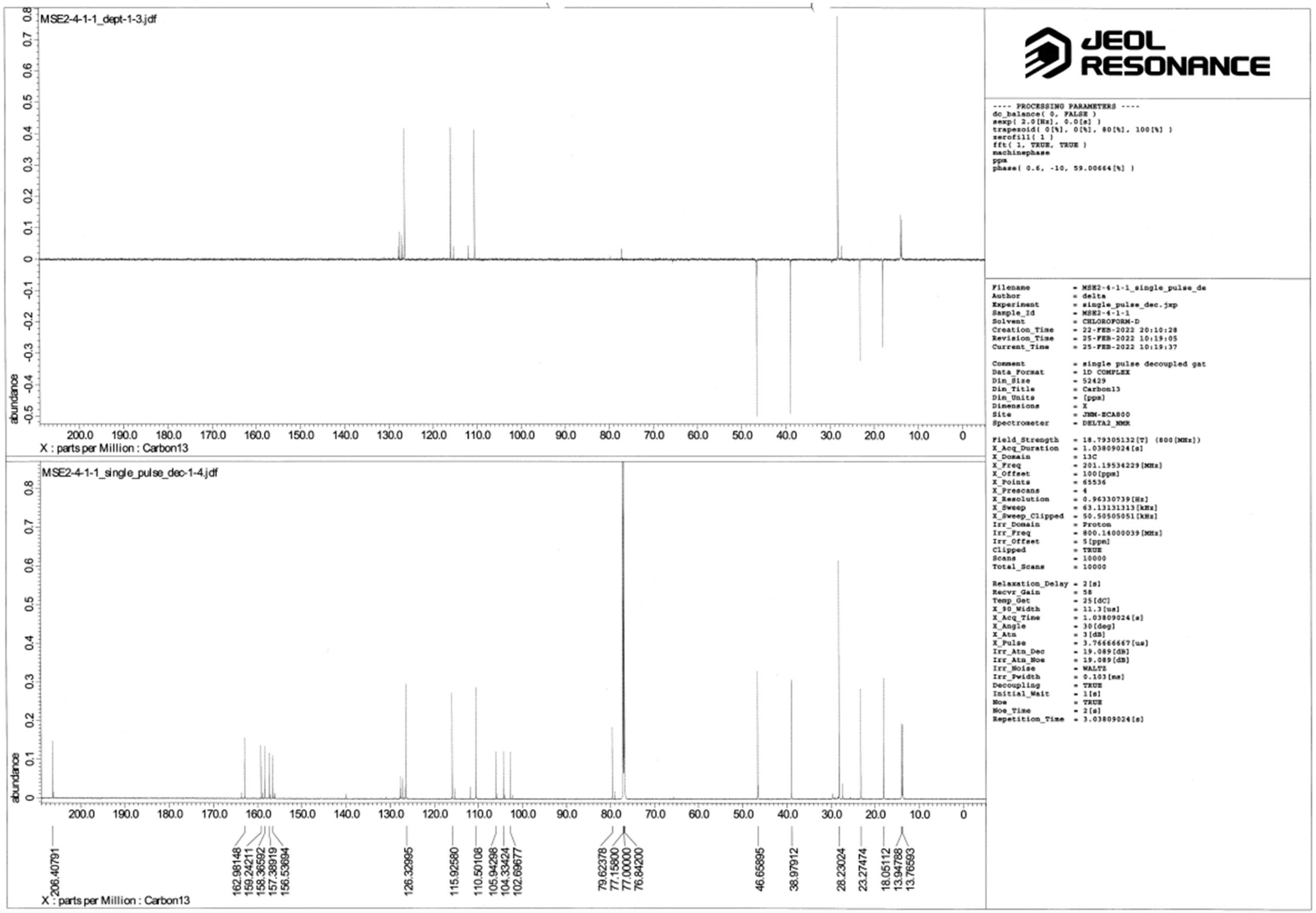

Figure S2. ^{13}C-NMR (200 MHz, $CDCl_3$) spectrum of mammeasin K (**1**)

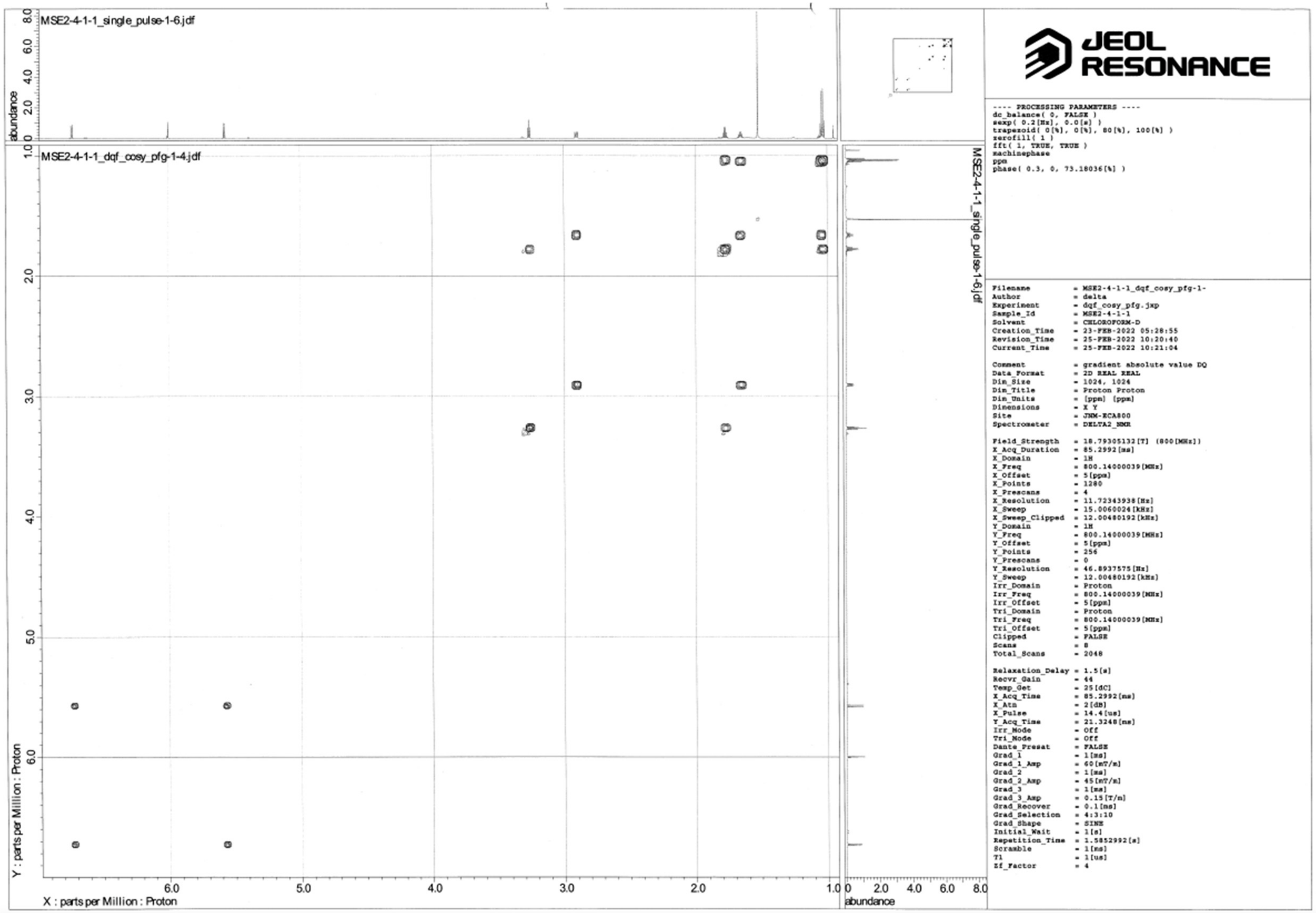

Figure S3. ^1H–^1H COSY spectrum of mammeasin K (**1**)

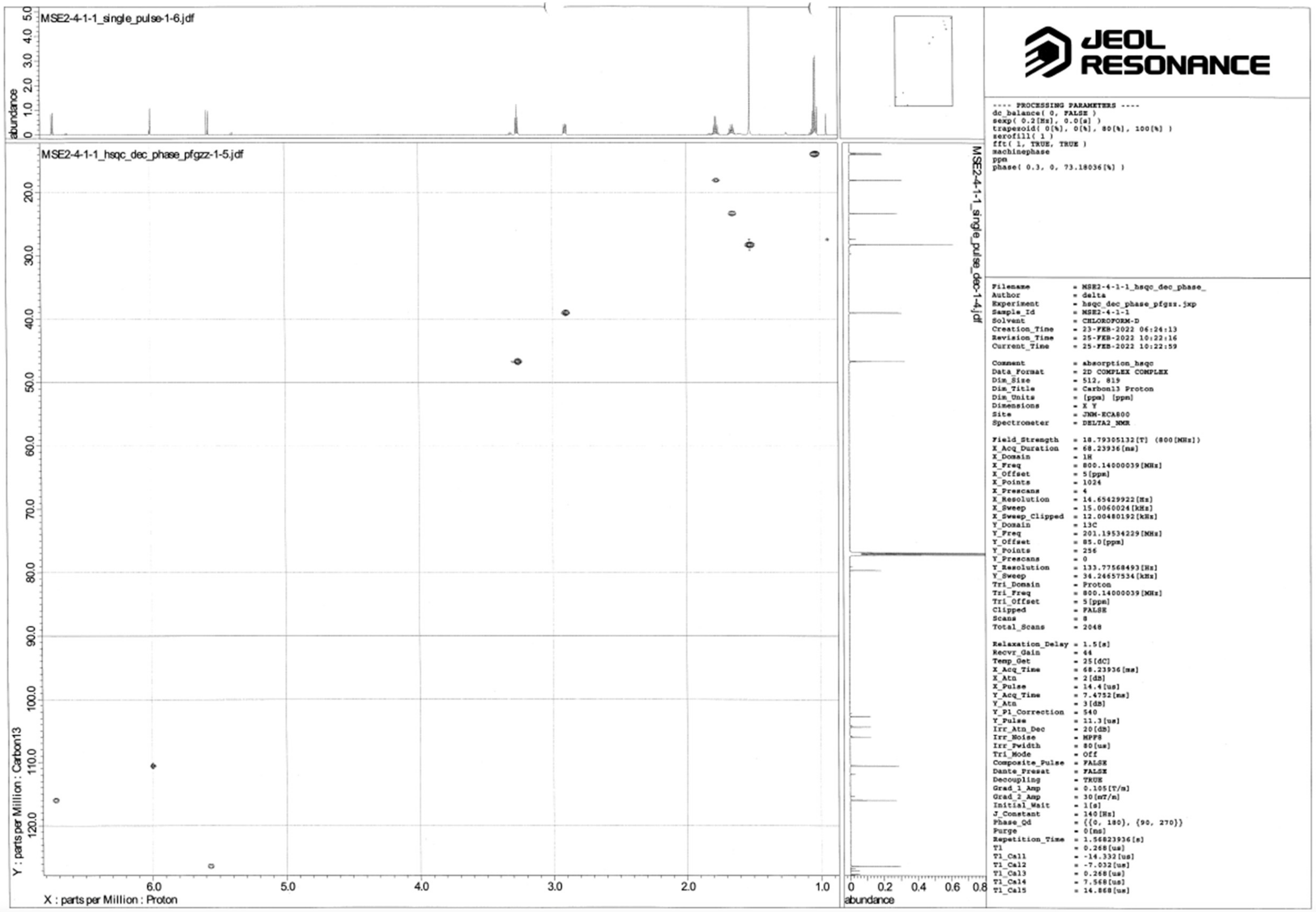

Figure S4. HSQC spectrum of mammeasin K (**1**)

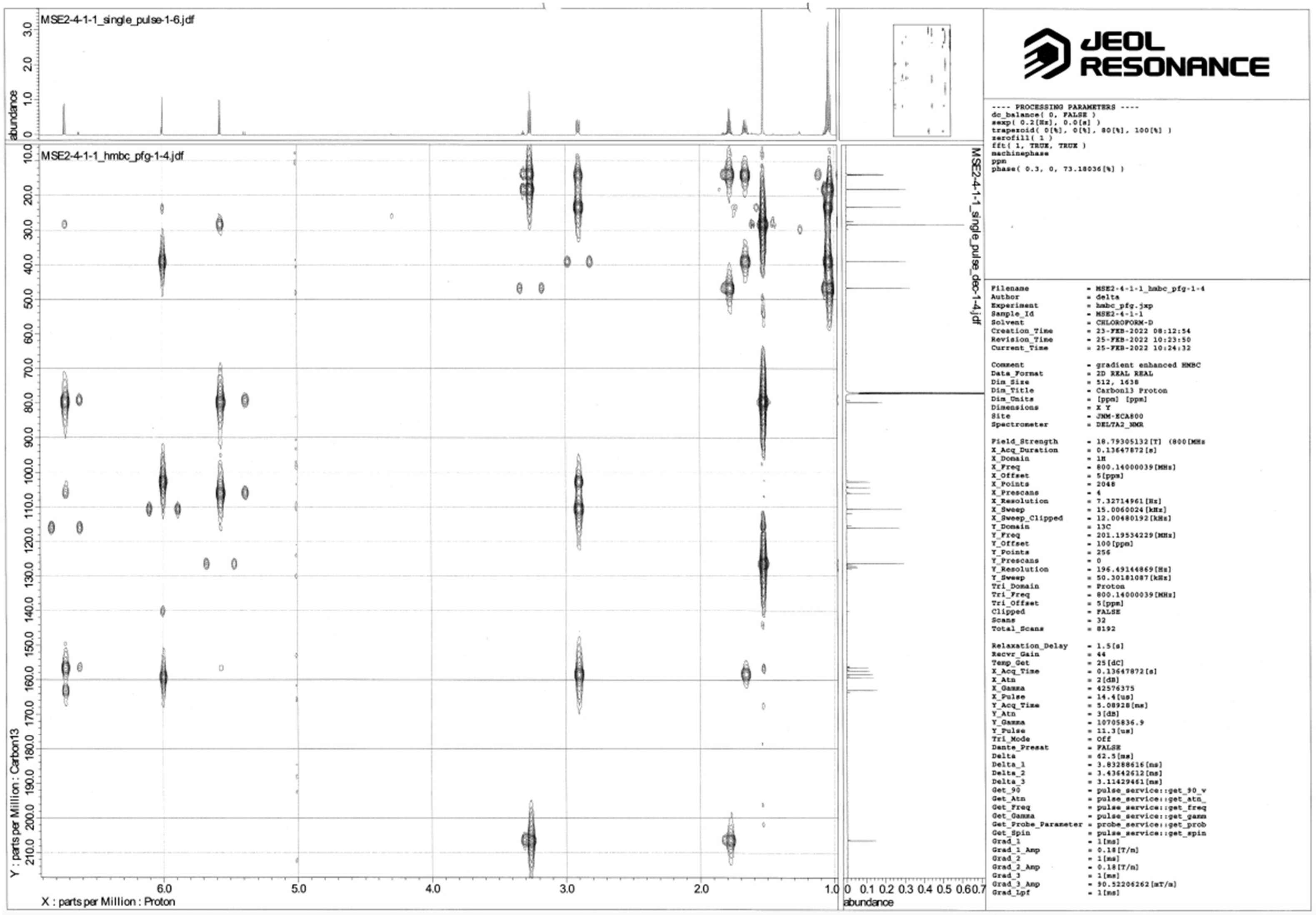

Figure S5. HMBC spectrum of mammeasin K (**1**)

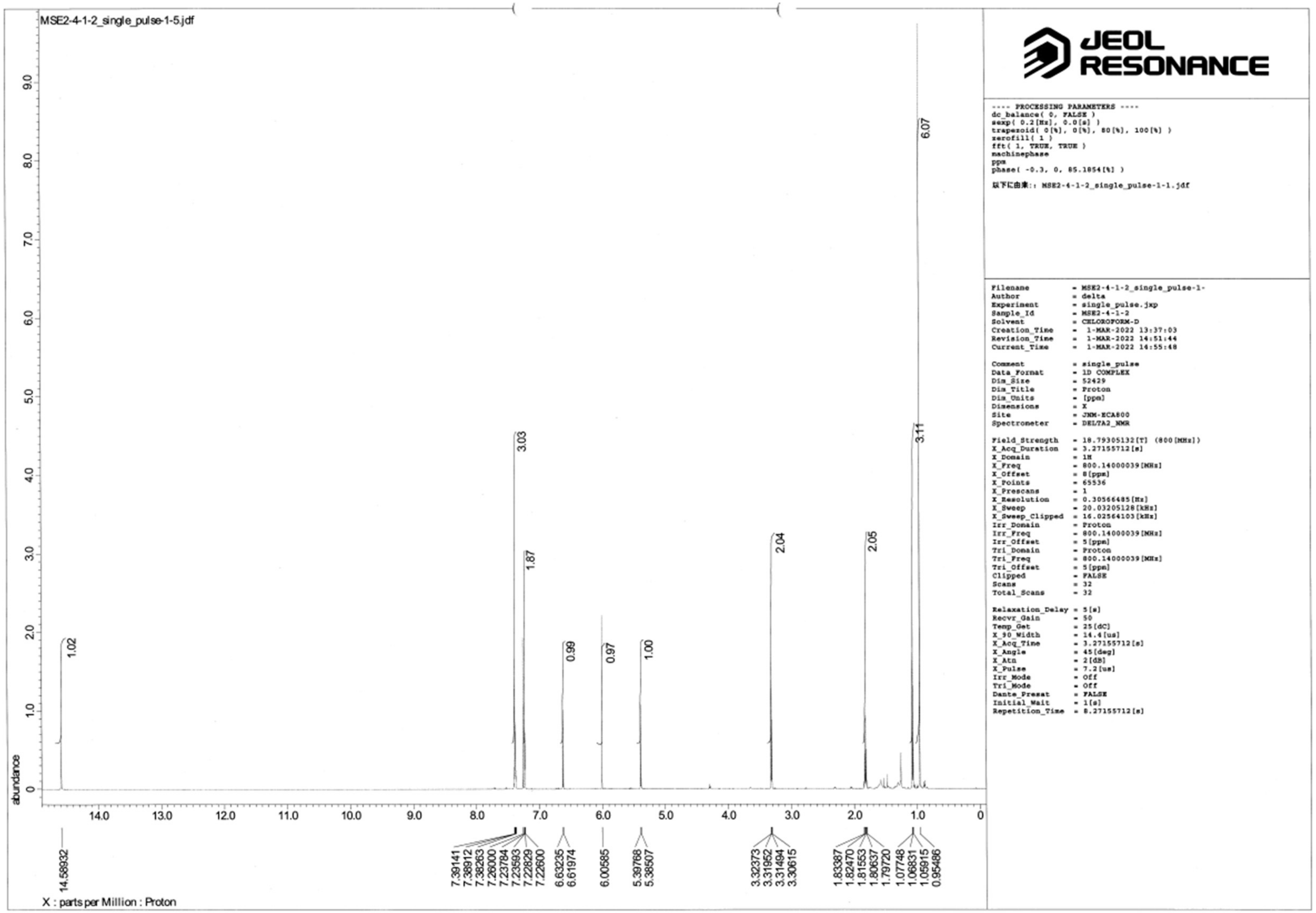

Figure S6. ¹H-NMR (800 MHz, $CDCl_3$) spectrum of mammeasin L (**2**)

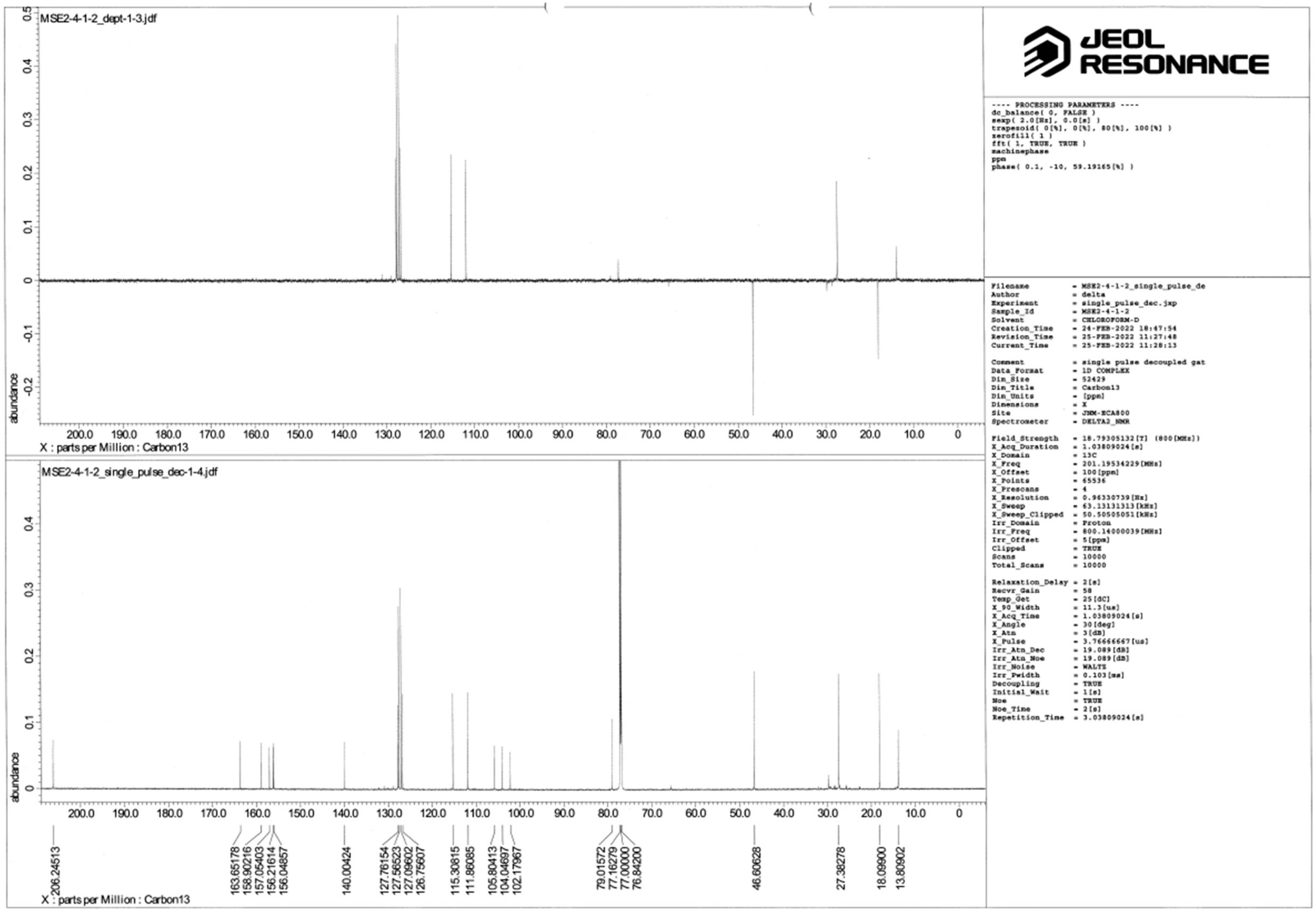

Figure S7. ^{13}C-NMR (200 MHz, $CDCl_3$) spectrum of mammeasin L (**2**)

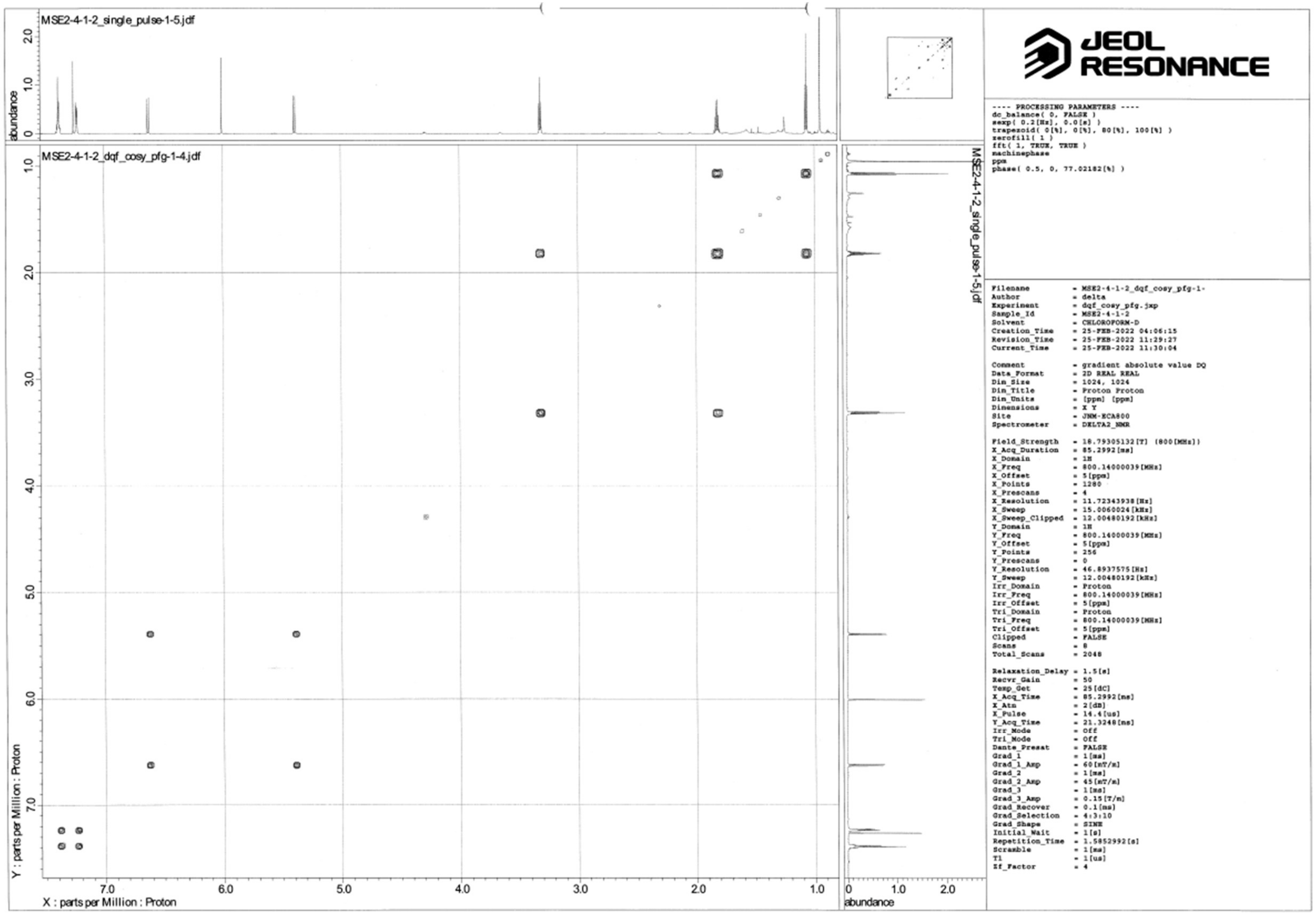

Figure S8. ^{1}H–^{1}H COSY spectrum of mammeasin L (**2**)

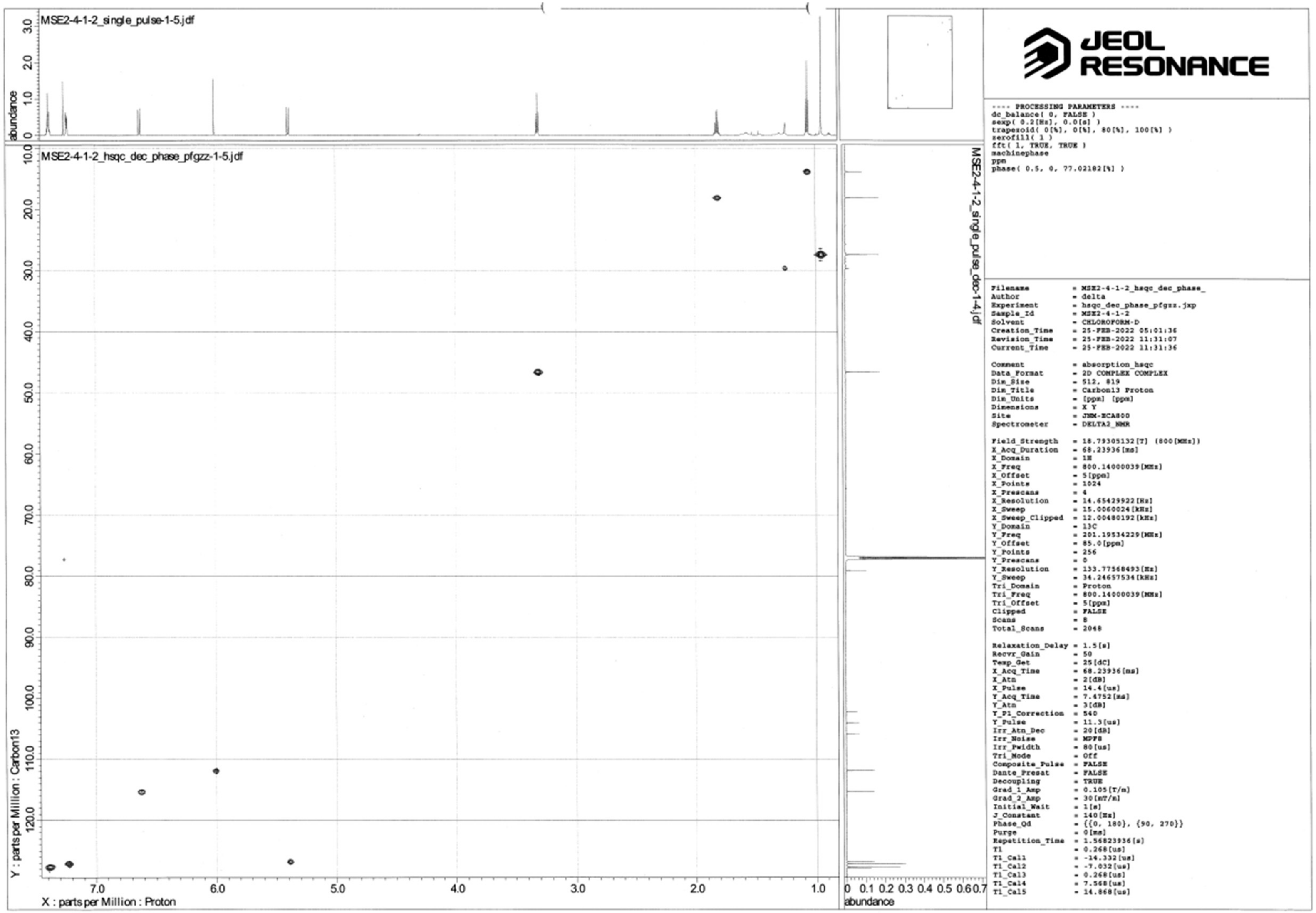

Figure S9. HSQC spectrum of mammeasin L (**2**)

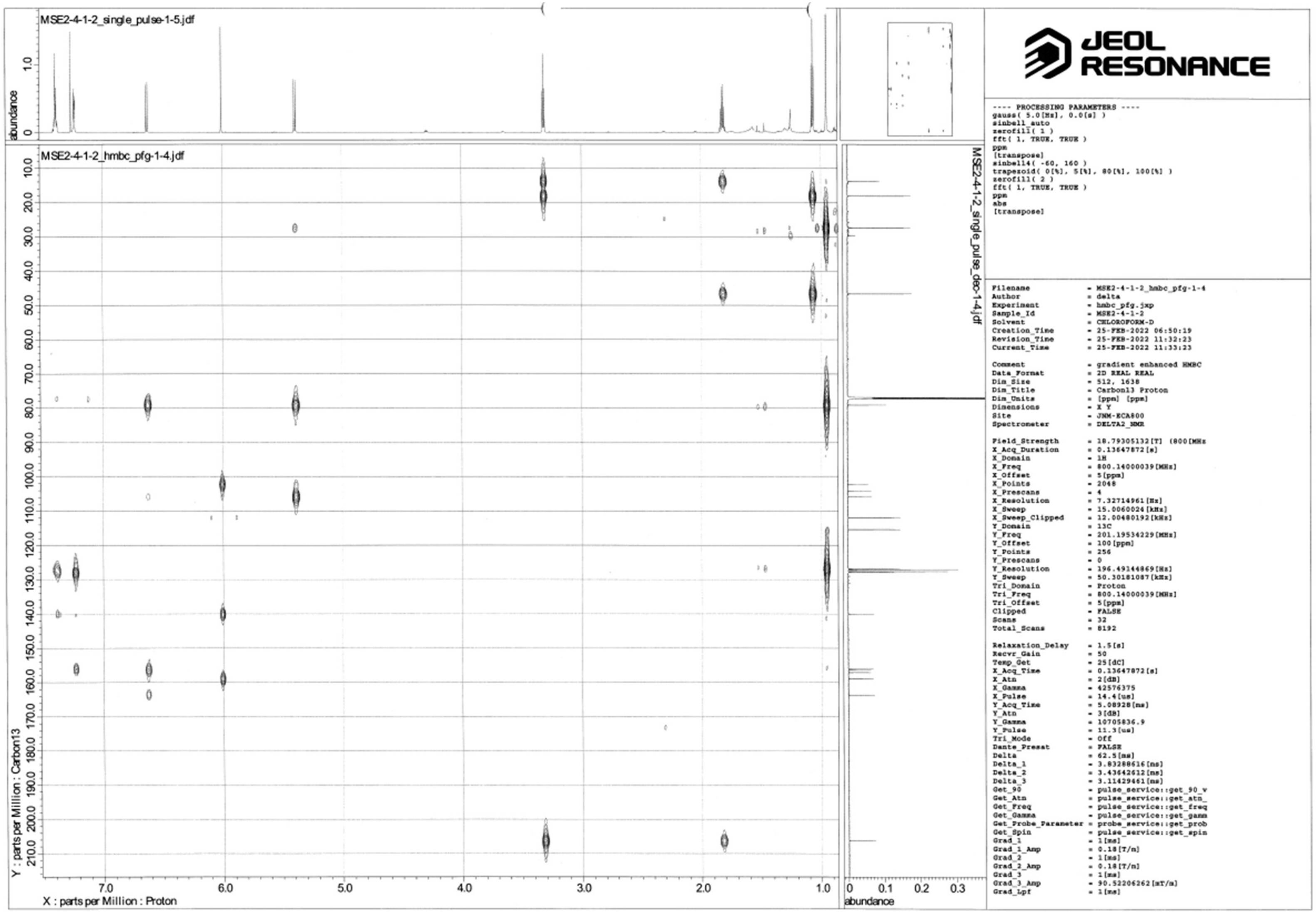

Figure S10. HMBC spectrum of mammeasin L (**2**)

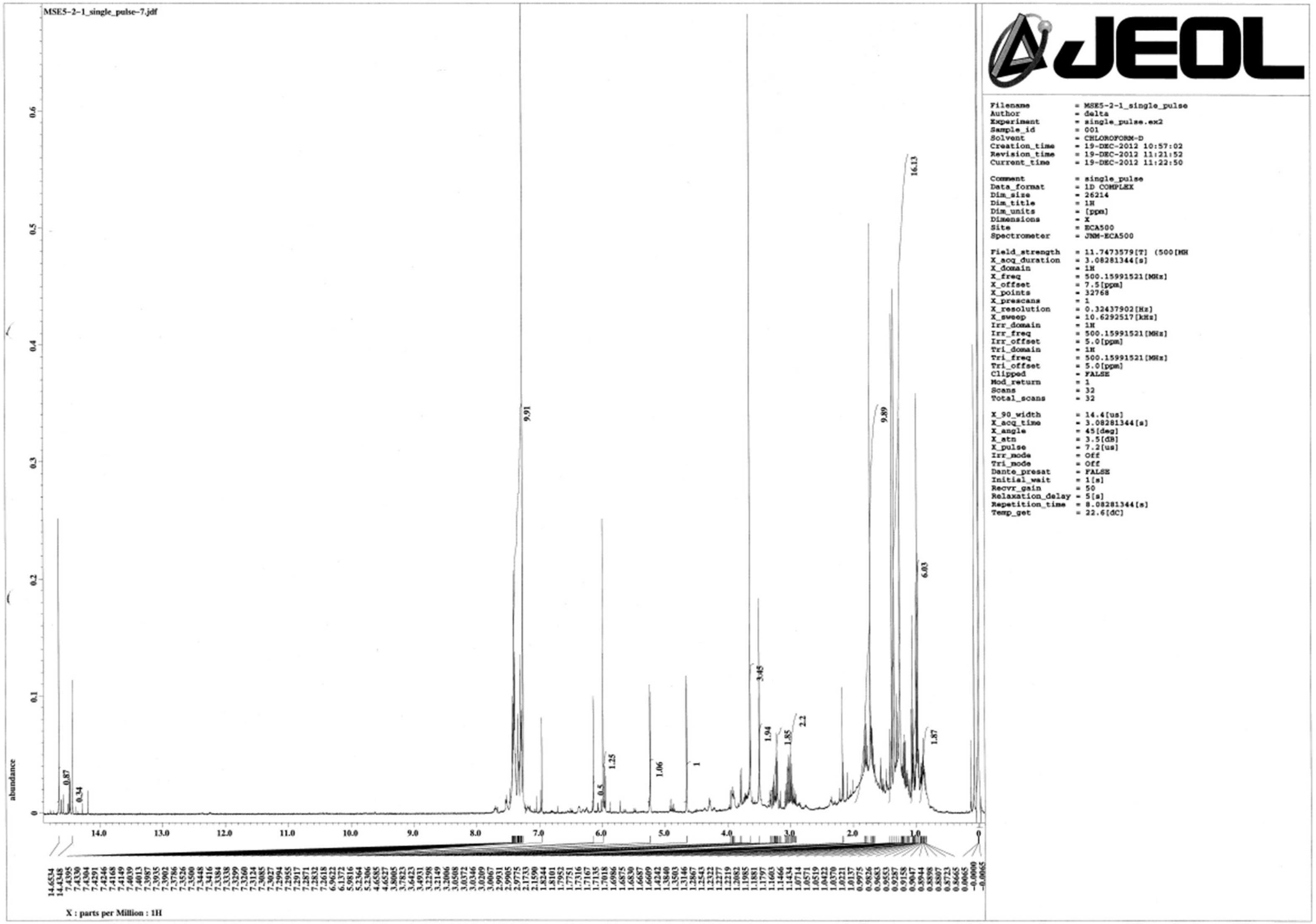

Figure S11. ^{1}H-NMR (500 MHz, $CDCl_3$) spectrum of mammeasin M (**3**)

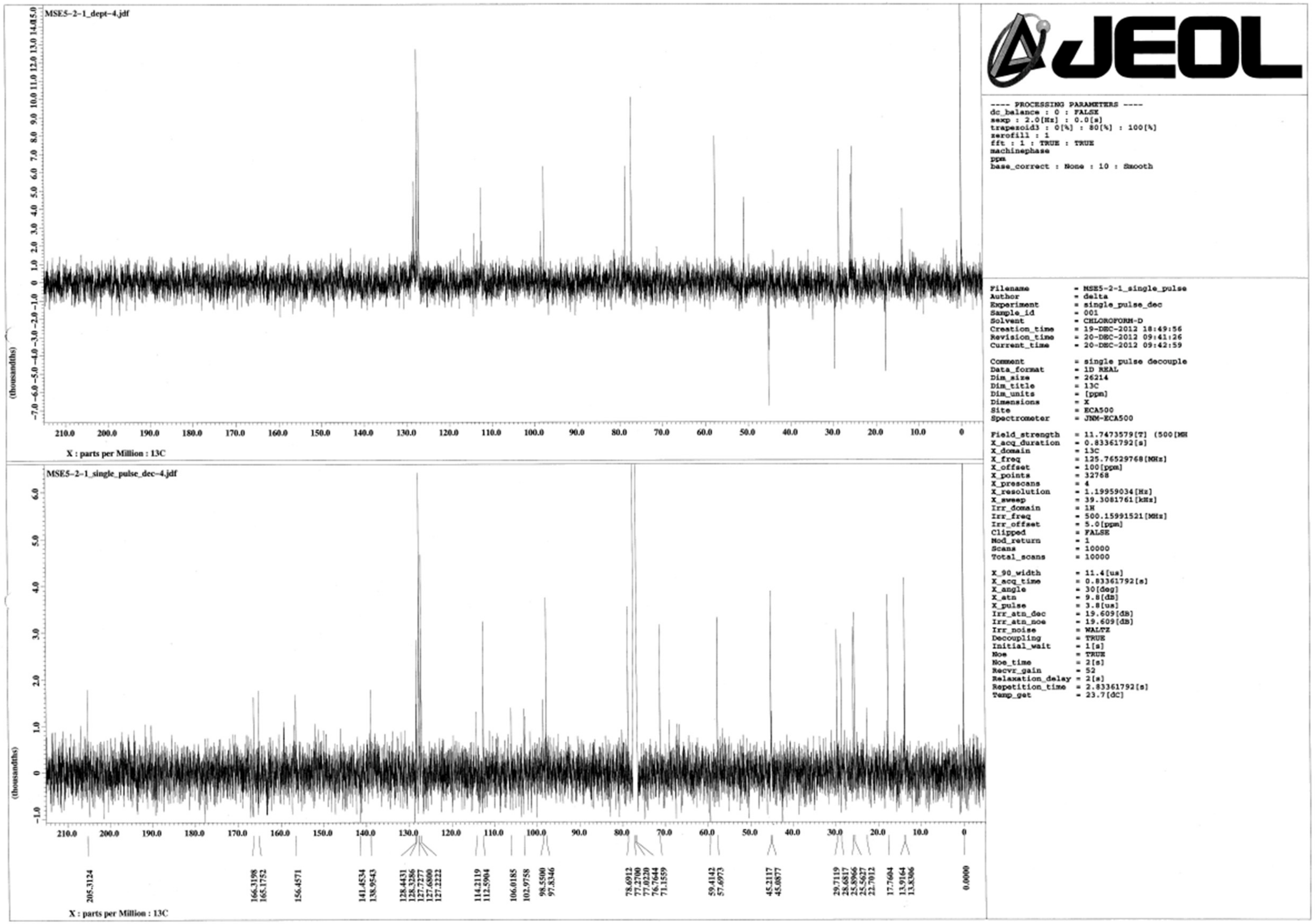

Figure S12. ^{13}C-NMR (125 MHz, $CDCl_3$) spectrum of mammeasin M (**3**)

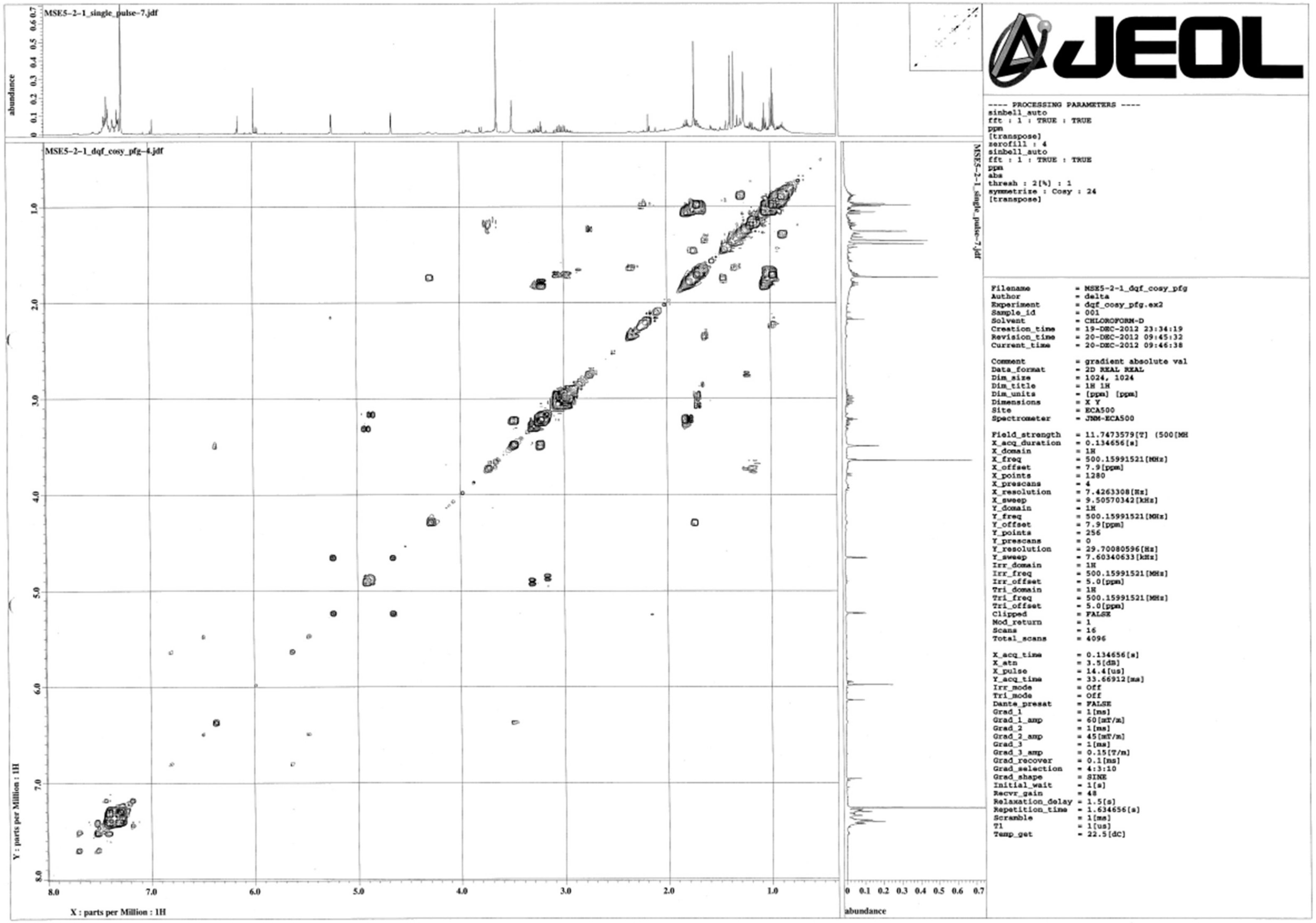

Figure S13. ^{1}H–^{1}H COSY spectrum of mammeasin M (**3**)

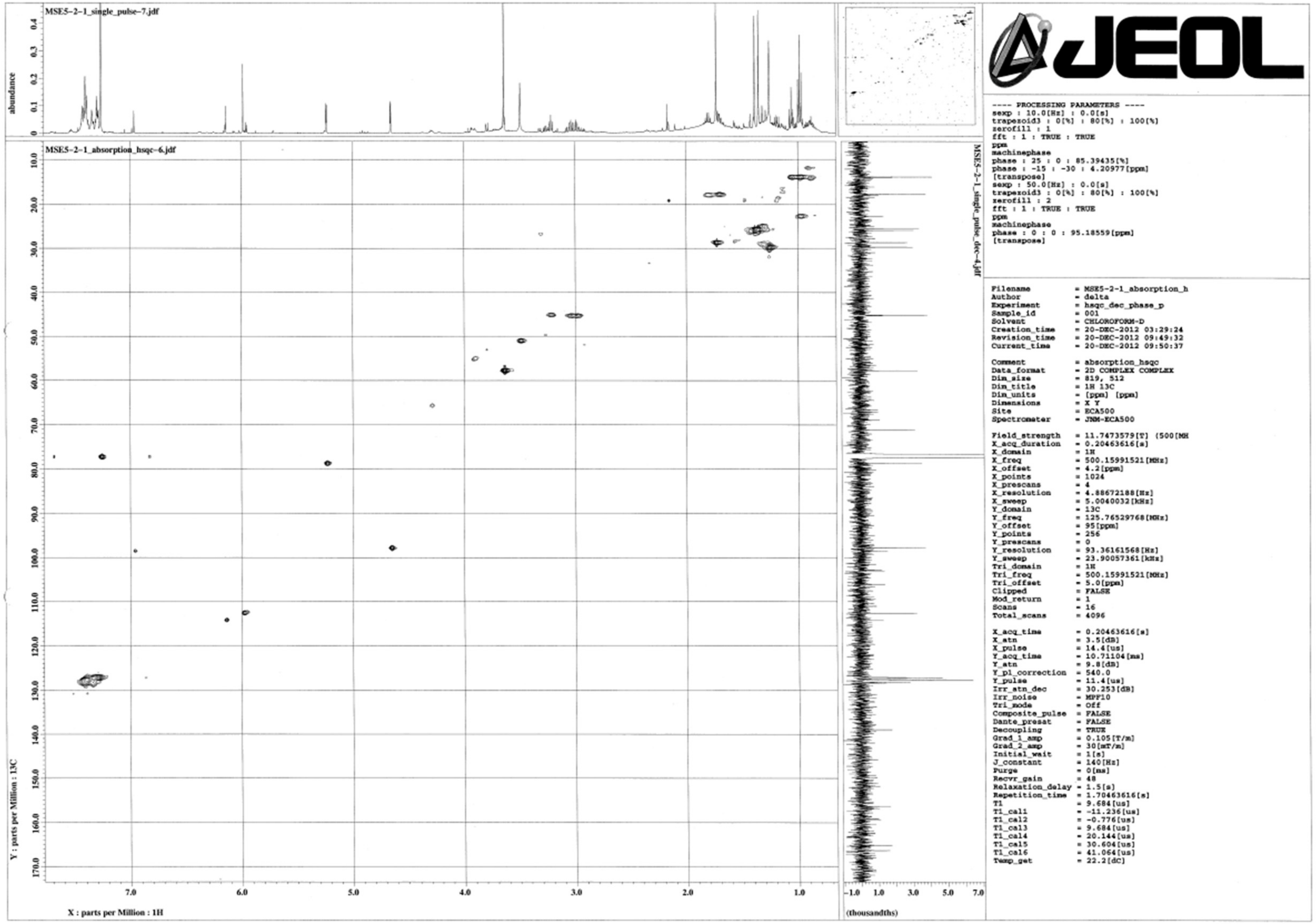

Figure S14. HSQC spectrum of mammeasin M (**3**)

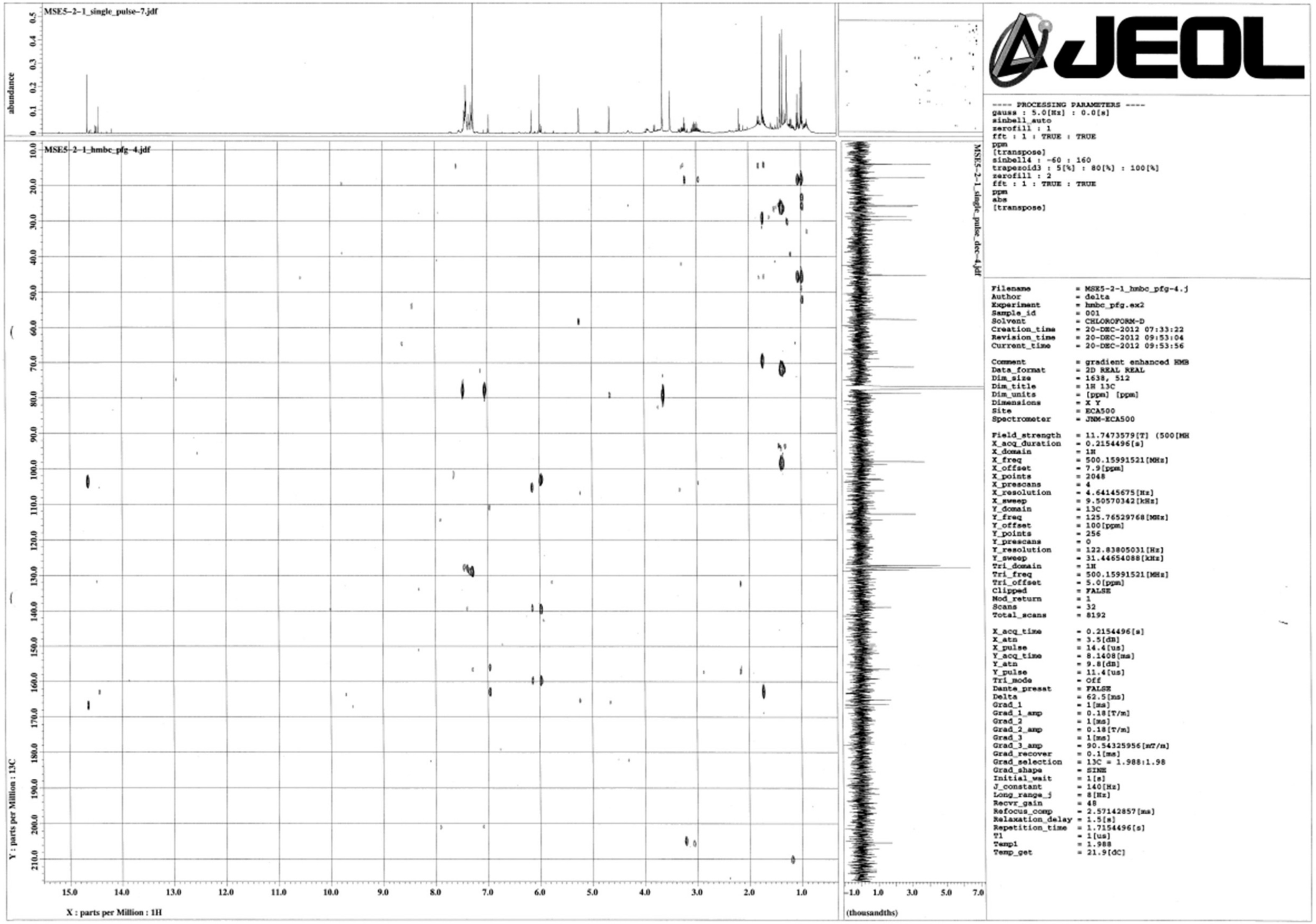

Figure S15. HMBC spectrum of mammeasin M (**3**)

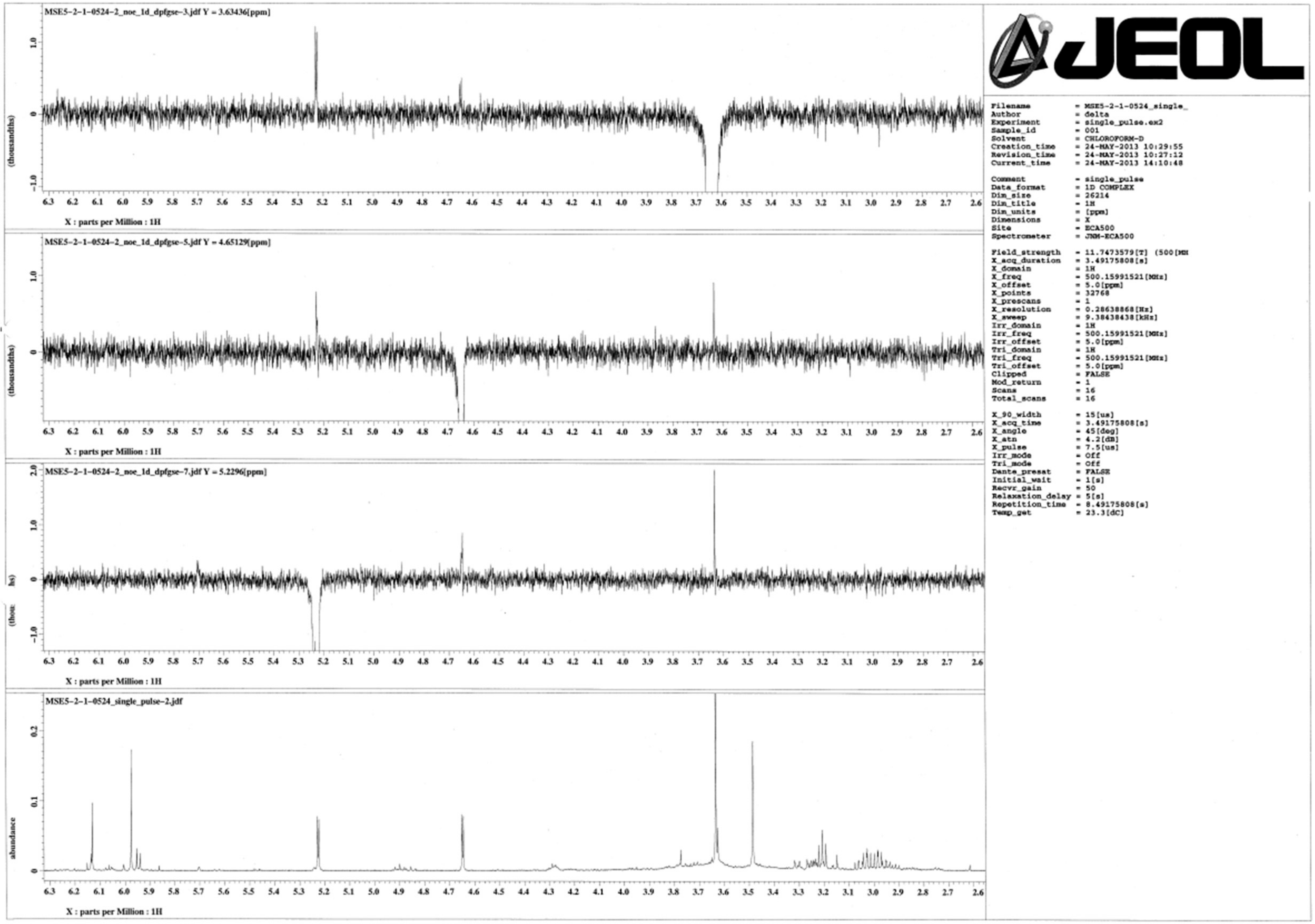

Figure S16. Difference NOE spectrum of mammeasin M (**3**)

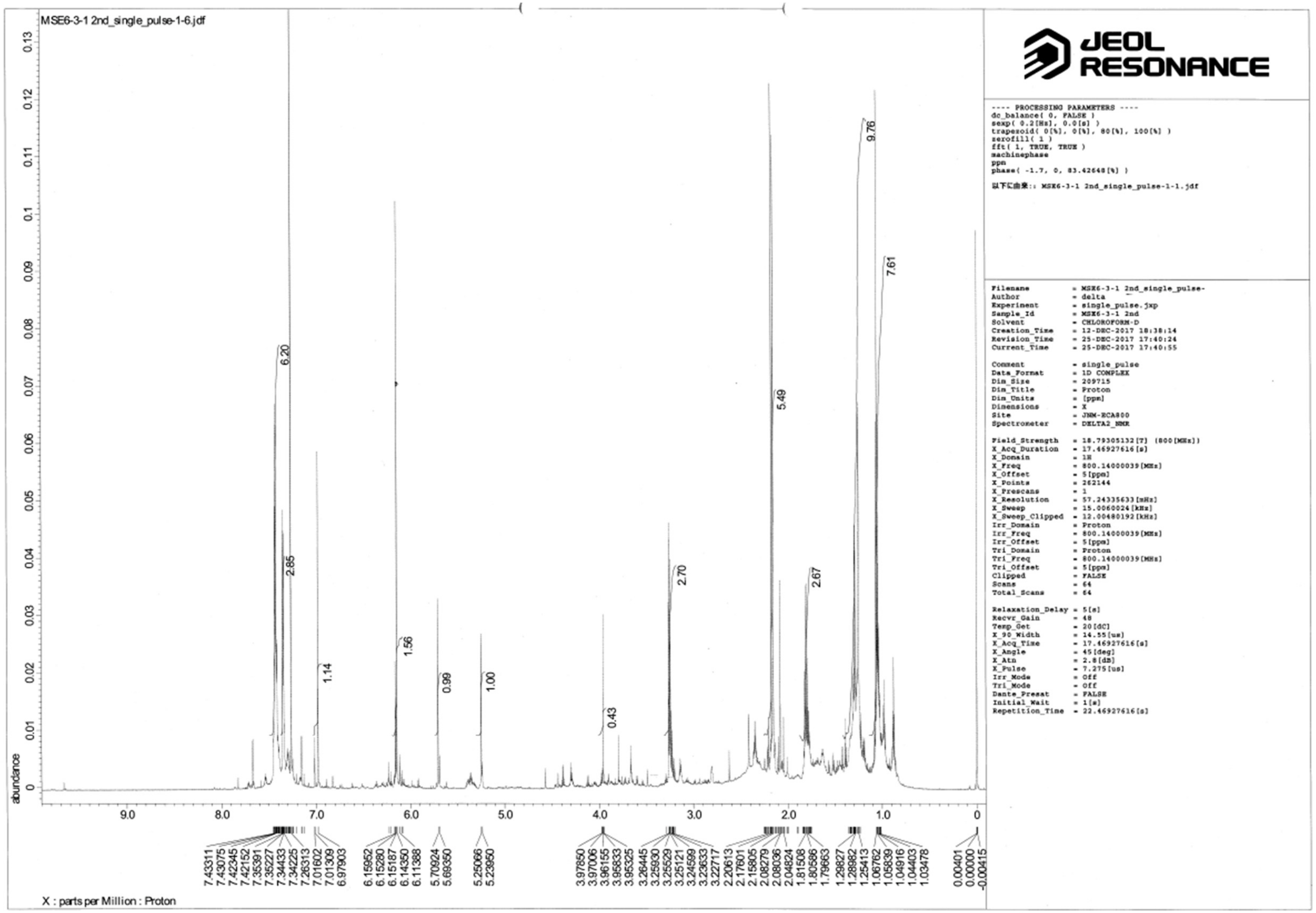

Figure S17. ^{1}H-NMR (800 MHz, $CDCl_3$) spectrum of mammeasin N (**4**)

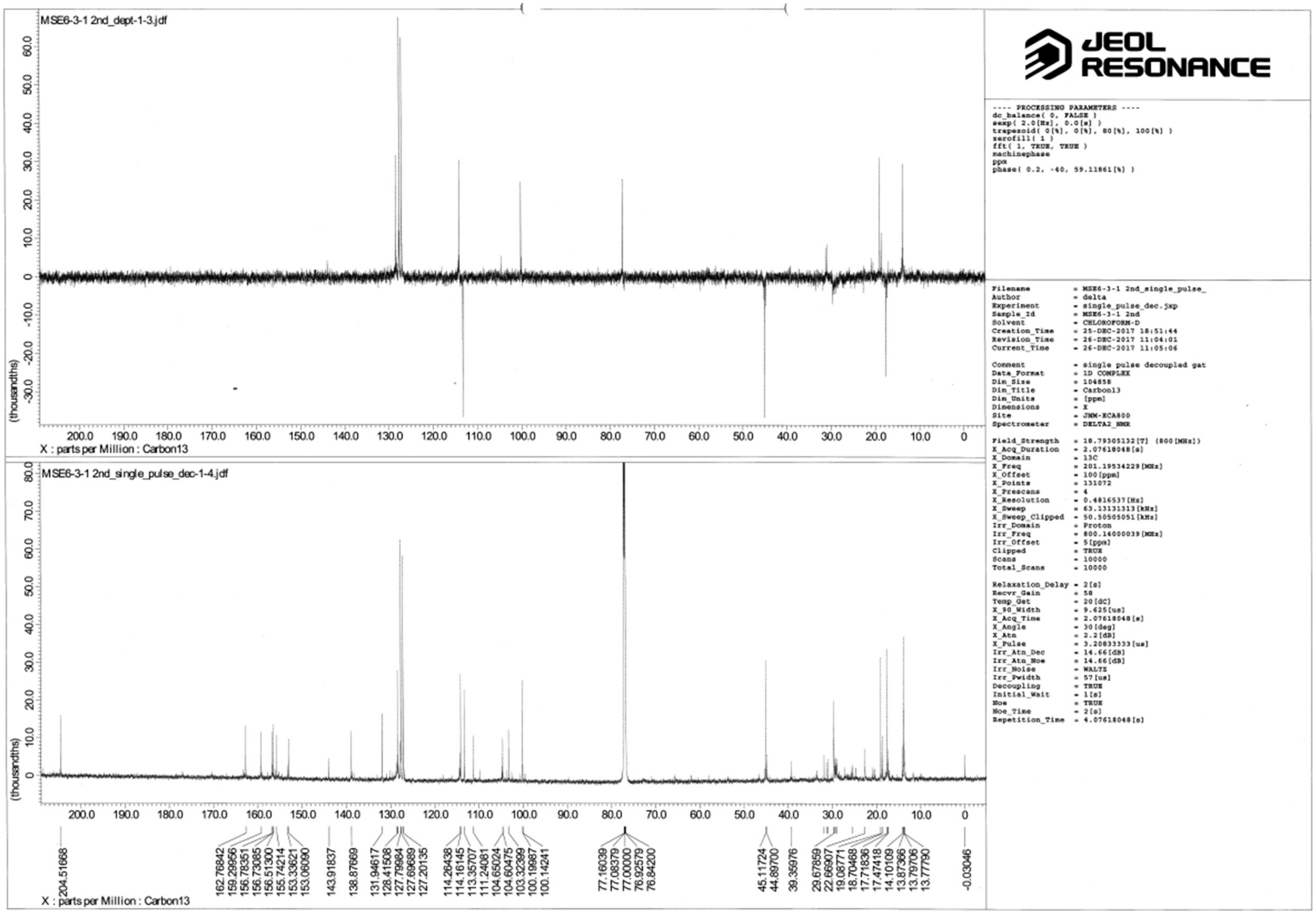

Figure S18. ^{13}C-NMR (200 MHz, $CDCl_3$) spectrum of mammeasin N (**4**)

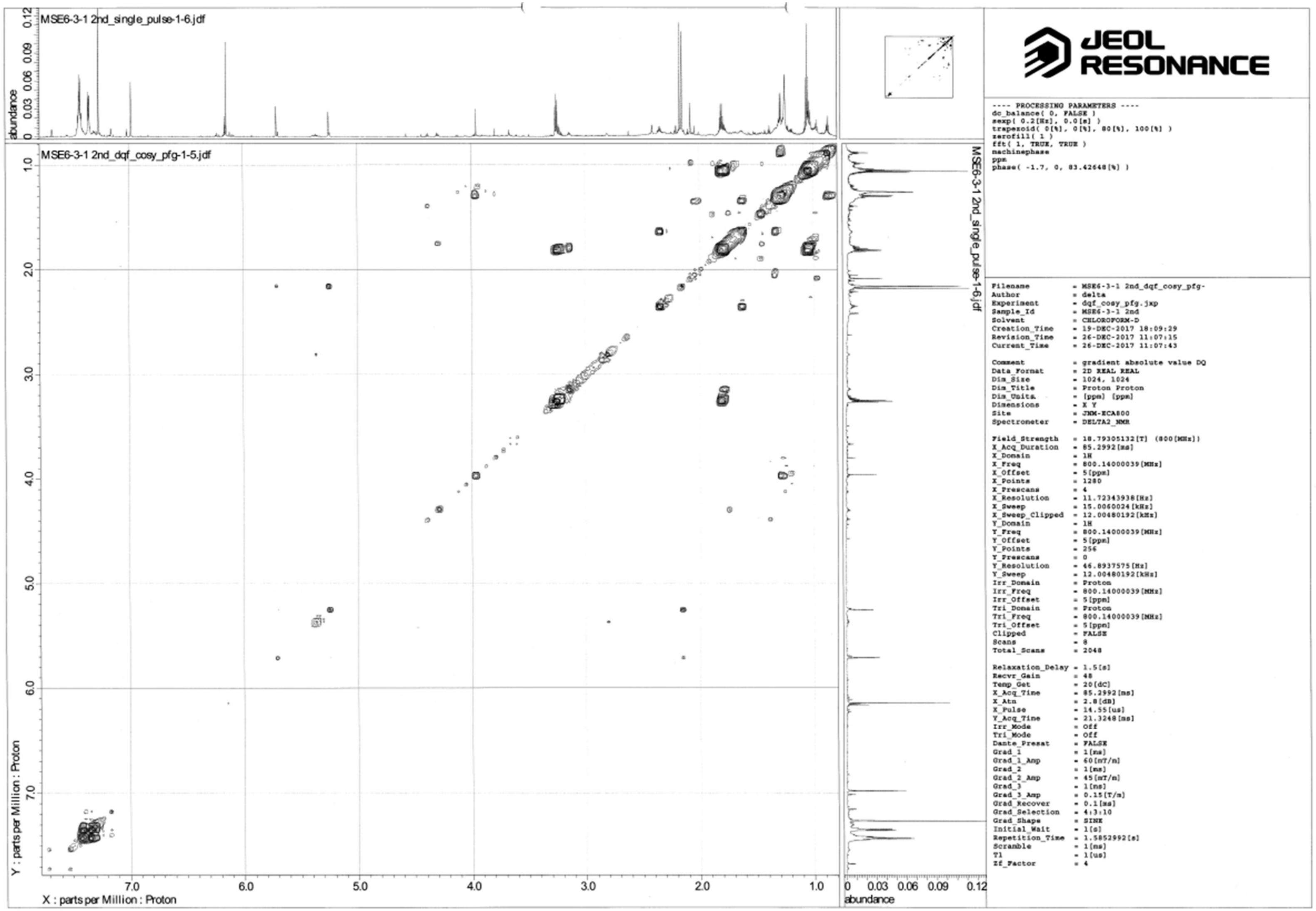

Figure S19. ^1H–^1H COSY spectrum of mammeasin N (**4**)

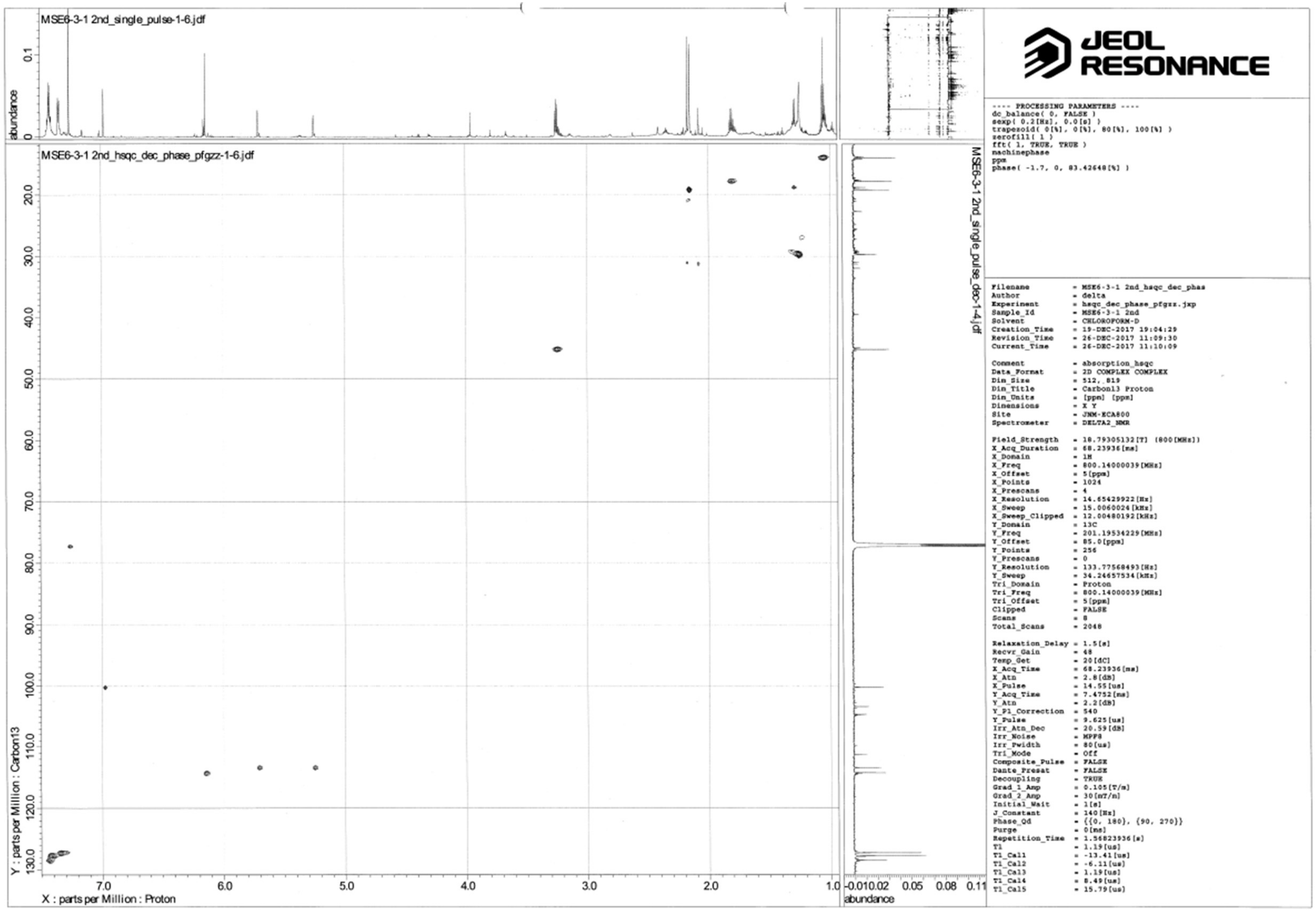

Figure S20. HSQC spectrum of mammeasin N (**4**)

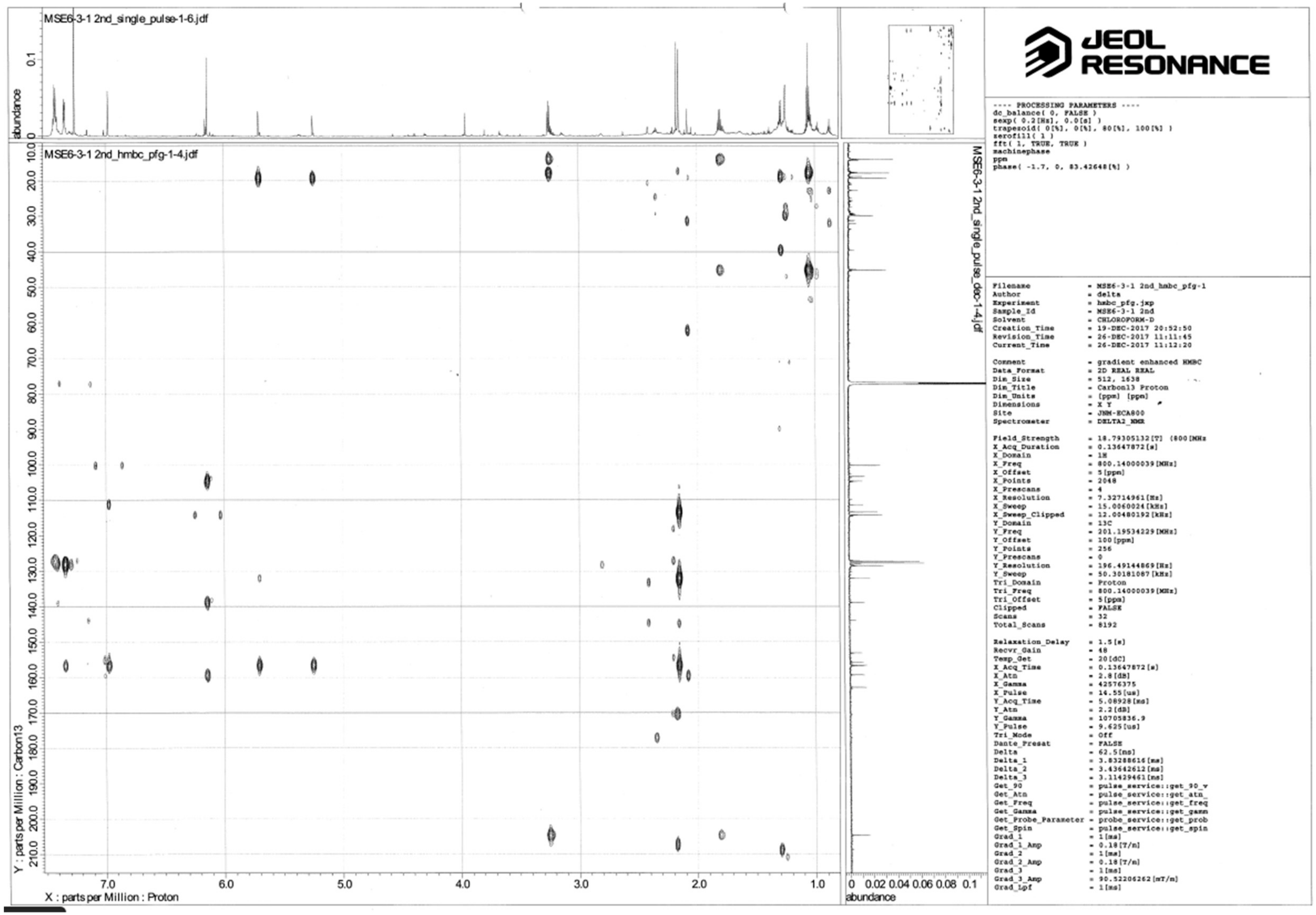

Figure S21. HMBC spectrum of mammeasin N (**4**)

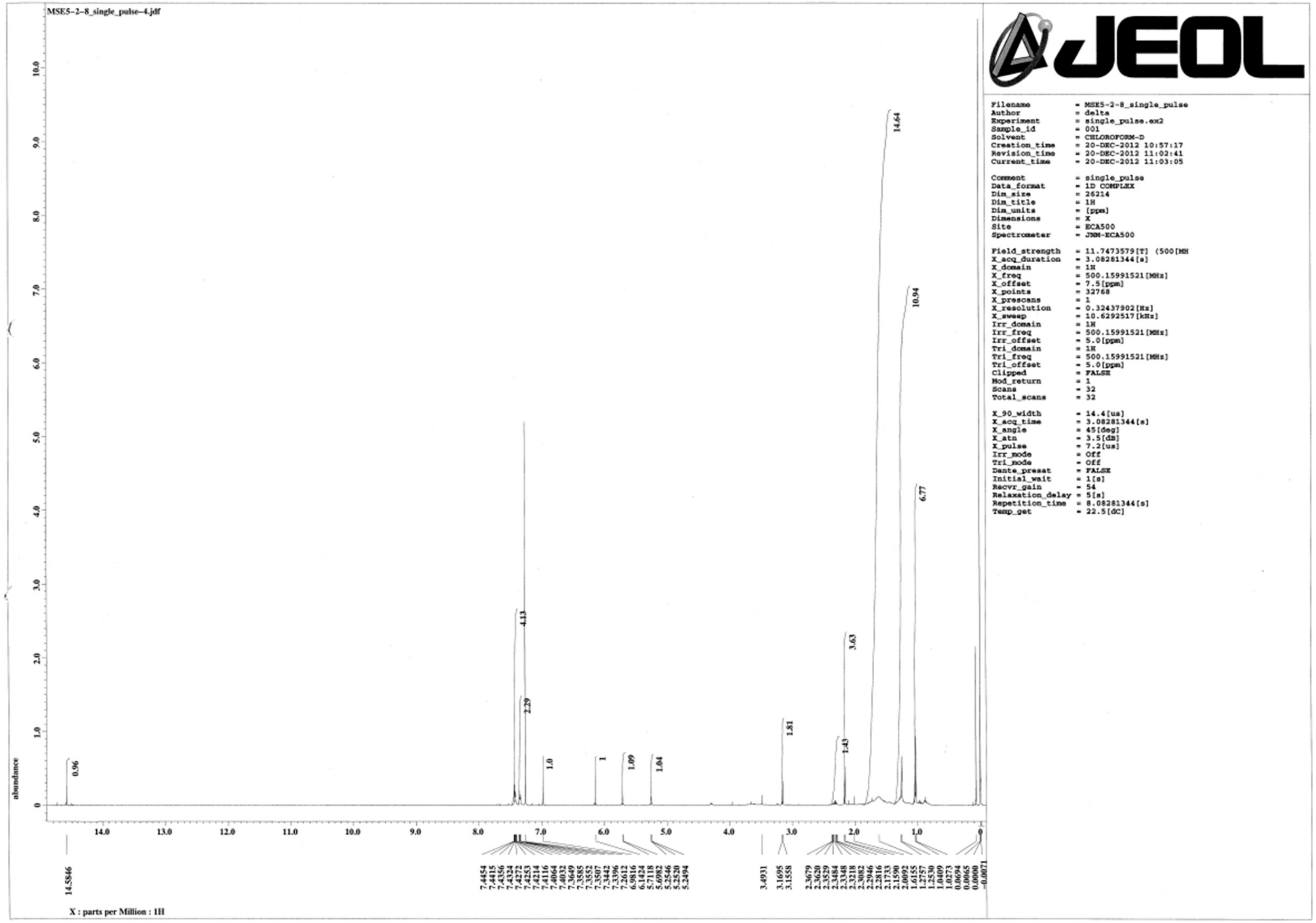

Figure S22. ^{1}H-NMR (500 MHz, $CDCl_3$) spectrum of mammeasin O (**5**)

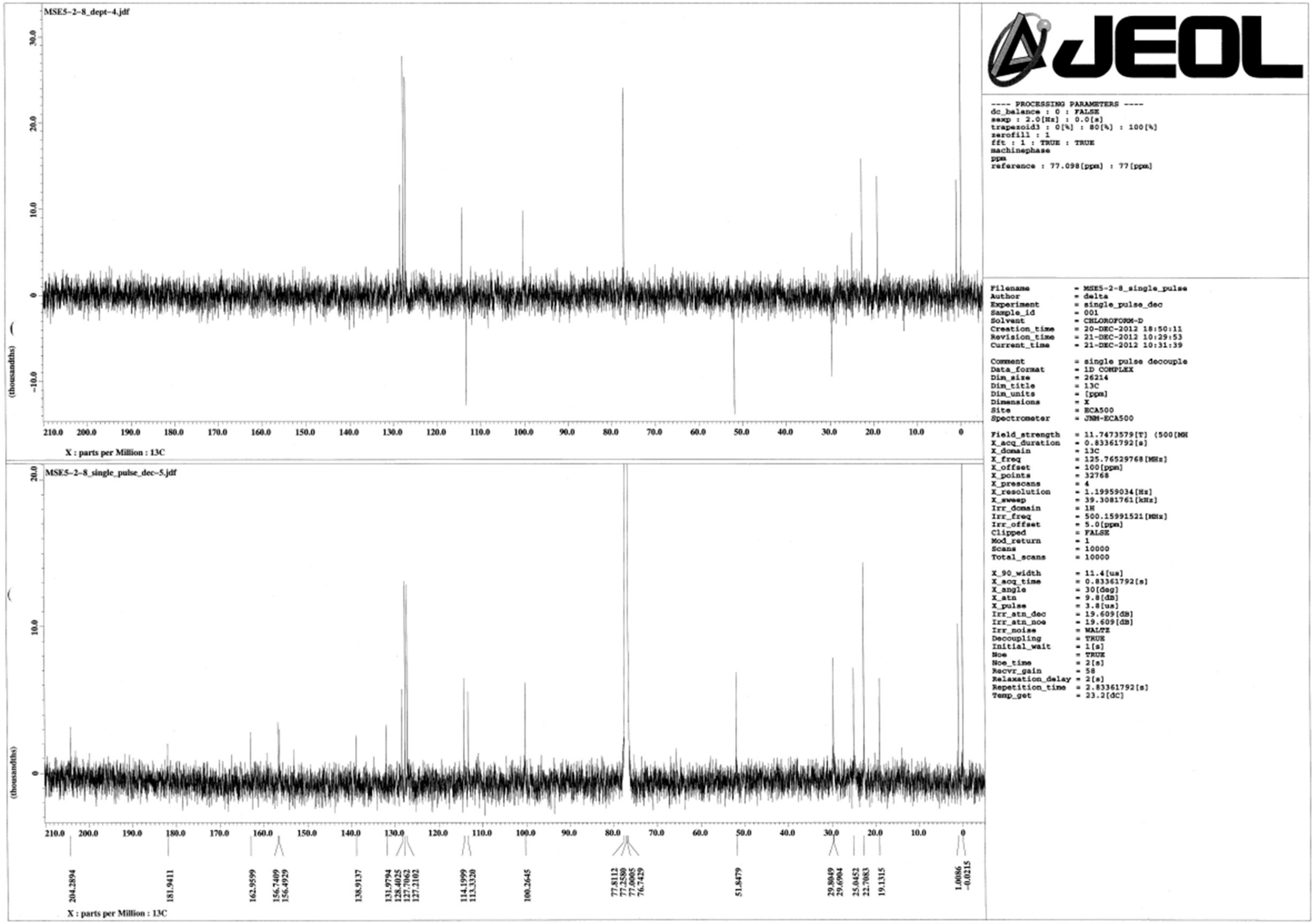

Figure S23. ^{13}C-NMR (125 MHz, $CDCl_3$) spectrum of mammeasin O (**5**)

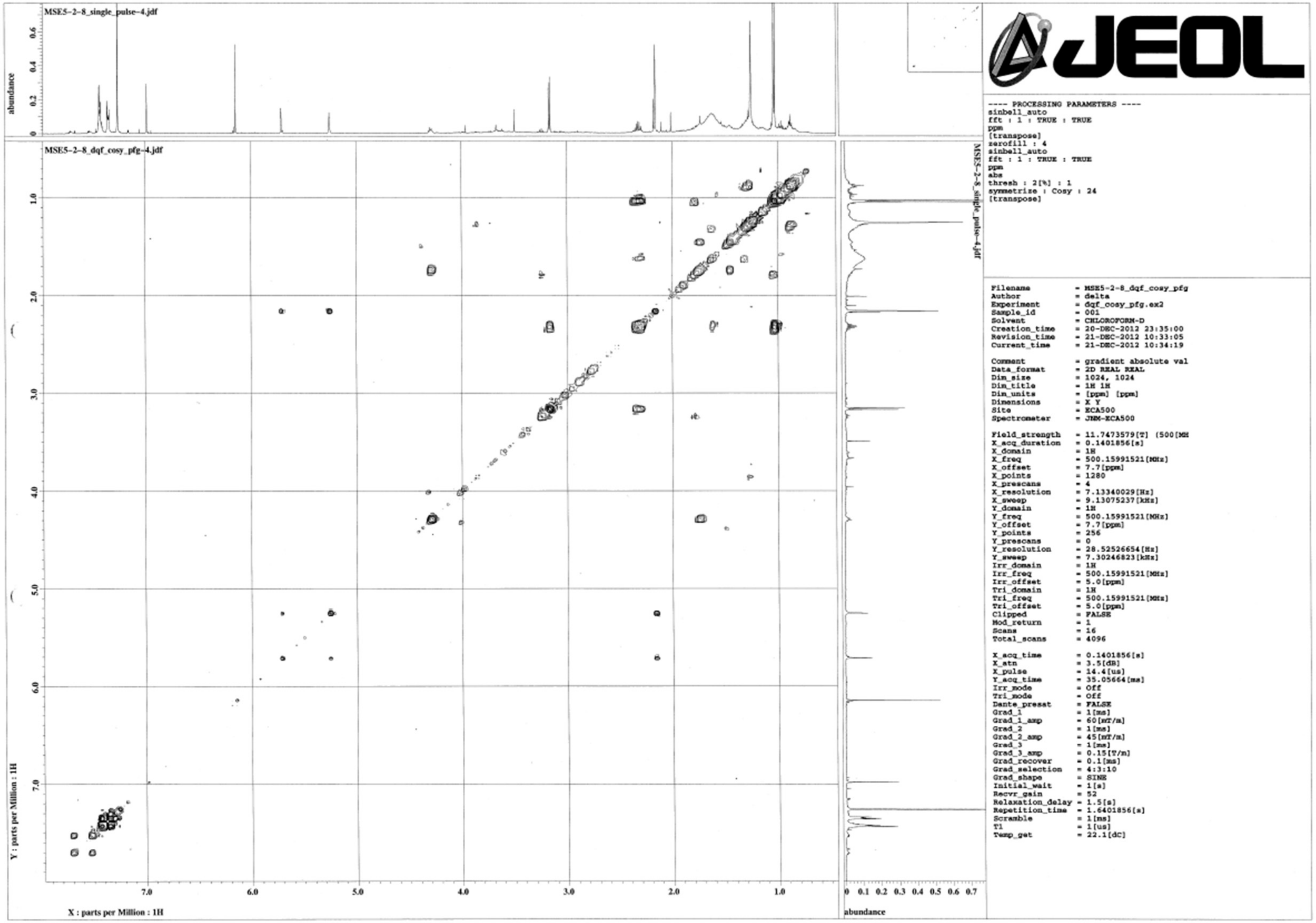

Figure S24. ^1H–^1H COSY spectrum of mammeasin O (**5**)

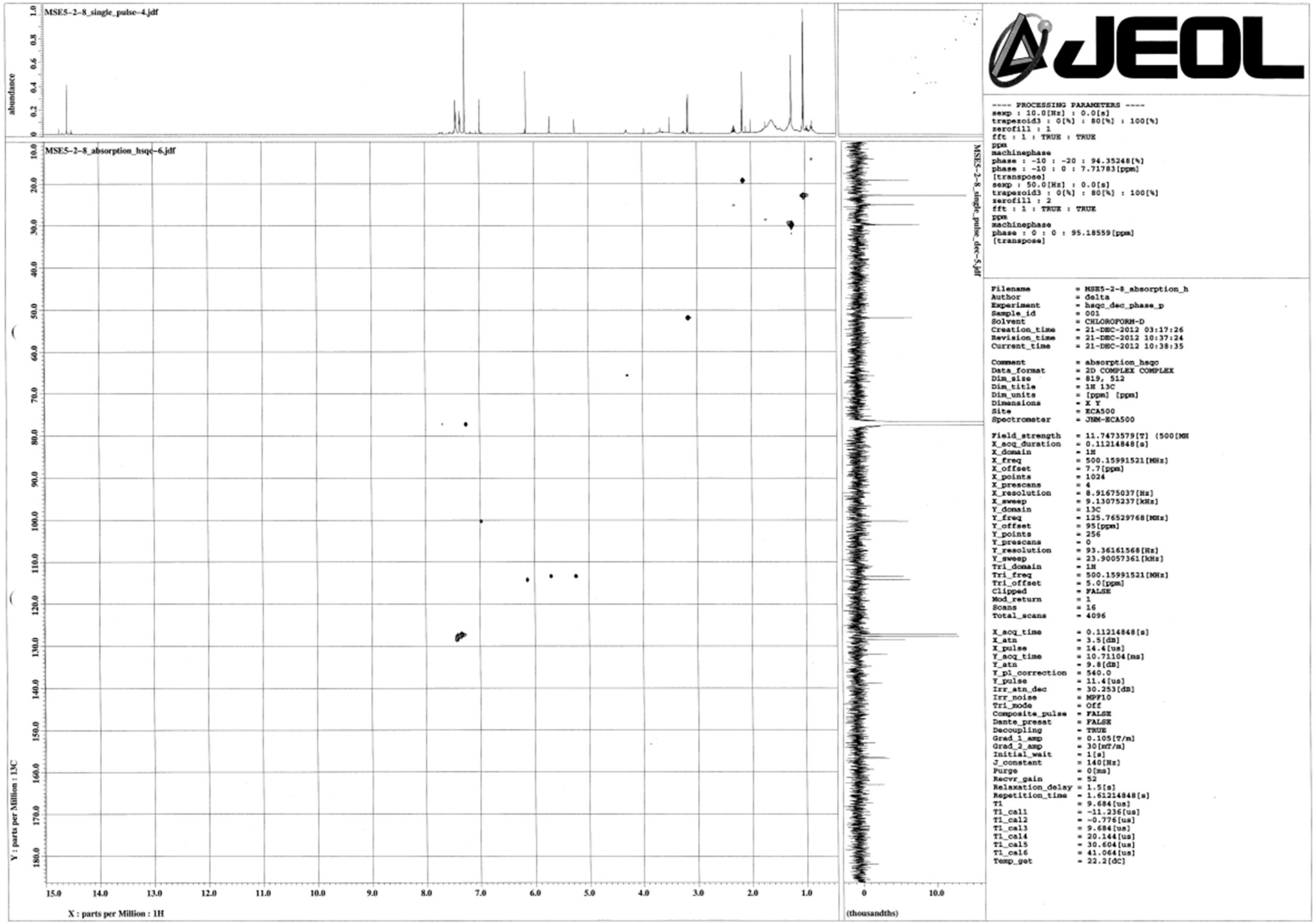

Figure S25. HSQC spectrum of mammeasin O (**5**)

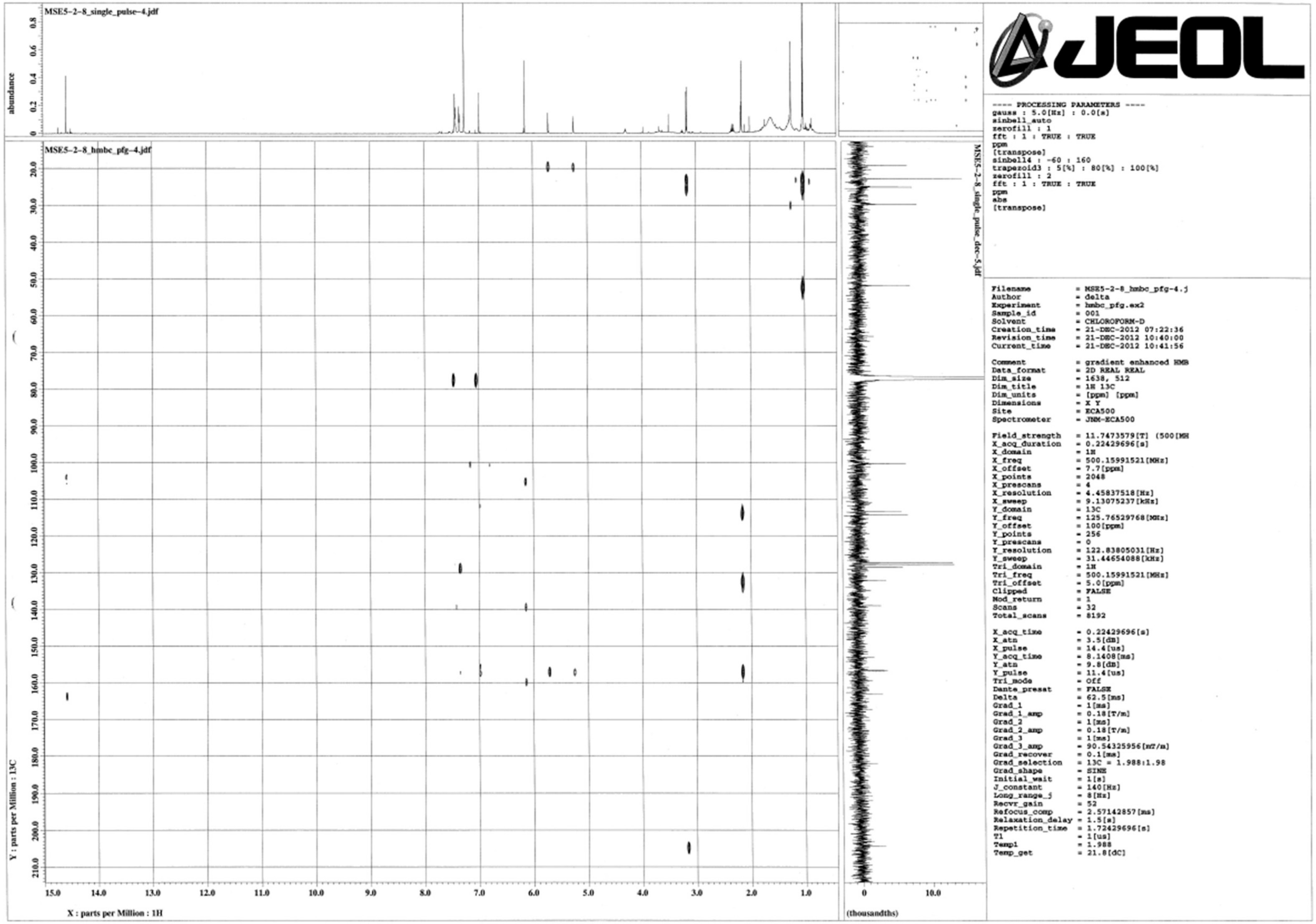

Figure S26. HMBC spectrum of mammeasin O (**5**)

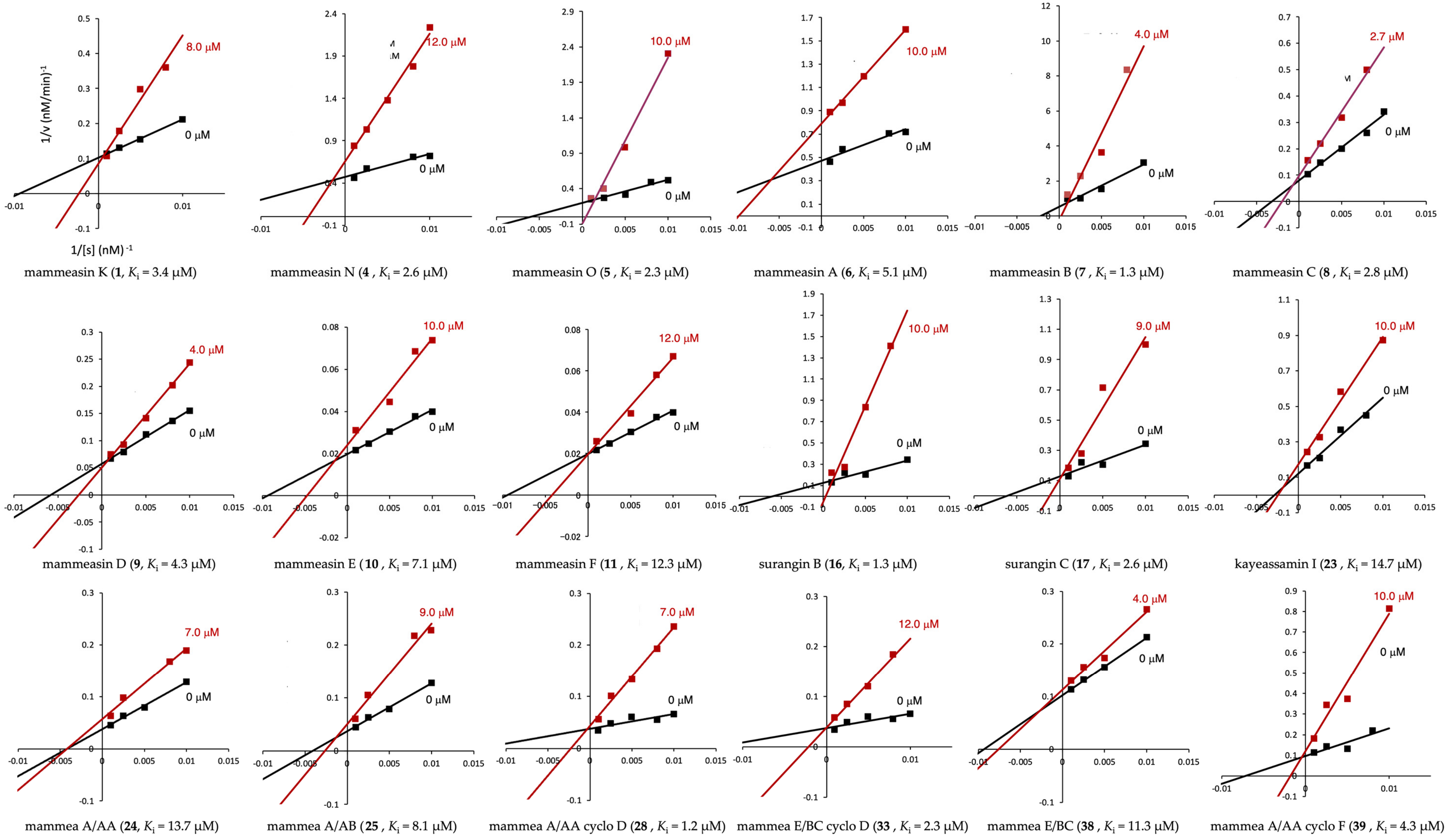

Figure S27. Lineweaver-Burk plots of the inhibition of human recombinant aromatase activity by **1**, **4**–**11**, **16**, **17**, **23**–**25**, **28**, **33**, **38**, and **39**
